# Prenatal Diagnosis and Individualized Perinatal Care in Apparently Nonmosaic Trisomy 9

**DOI:** 10.1155/crig/7719810

**Published:** 2026-10-11

**Authors:** Hitoshi Isohata, Yoshihiro Yoshimura, Yu Yamazaki, Hiroyuki Goto, Kyoko Hattori, Takao Shimaoka, Fumio Takada, Daigo Ochiai

**Affiliations:** ^1^ Department of Obstetrics and Gynecology Kitasato University School of Medicine Sagamihara Kanagawa Japan kitasato-u.ac.jp; ^2^ Department of Medical Genetics and Genomics Kitasato University Graduate School of Medical Sciences Sagamihara Kanagawa Japan kitasato-u.ac.jp

**Keywords:** multidisciplinary management, nonmosaic Trisomy 9, perinatal palliative care, phenotype, prenatal diagnosis

## Abstract

**Background:**

Nonmosaic Trisomy 9 is a rare chromosomal abnormality, accounting for approximately 2.2%–2.7% of spontaneous abortions caused by autosomal trisomies, and is characterized by a heterogeneous multisystem phenotype and an extremely poor prognosis. Prenatal imaging may demonstrate severe multisystem abnormalities; however, these findings are not specific to Trisomy 9, making cytogenetic testing essential for definitive diagnosis. We report a case of prenatally diagnosed apparently nonmosaic Trisomy 9 in which diagnosis enabled individualized perinatal care.

**Case:**

A 44‐year‐old multiparous woman was referred to our hospital at 25 weeks’ gestation because of severe fetal growth restriction and multiple structural anomalies. Amniocentesis at 28 weeks’ gestation demonstrated 47,XY,+9 in all 100 examined colonies, consistent with apparently nonmosaic Trisomy 9. Following multidisciplinary counseling and shared decision‐making, an individualized birth plan was developed. The family wished to avoid aggressive life‐prolonging interventions while prioritizing the opportunity to meet their infant alive; therefore, fetal monitoring, cesarean delivery for fetal indications, and mask ventilation without further resuscitation were incorporated into the plan. Emergency cesarean delivery was performed at 36 weeks and 2 days for nonreassuring fetal status. The infant died approximately 9 h after birth while receiving family‐centered palliative care.

**Conclusion:**

This case highlights the clinical importance of definitive prenatal cytogenetic diagnosis in apparently nonmosaic Trisomy 9, enabling phenotype‐informed prognostic counseling and individualized perinatal management.

## 1. Introduction

Nonmosaic Trisomy 9 is a rare chromosomal abnormality, accounting for approximately 2.2%–2.7% of spontaneous abortions caused by autosomal trisomies [1], and is associated with an extremely poor prognosis. Its phenotype is heterogeneous and involves multiple organ systems, with commonly reported abnormalities affecting the craniofacial, central nervous, cardiovascular, renal/genitourinary, and musculoskeletal systems [2, 3]. Most affected fetuses die in utero or during the early neonatal period [2, 3]. Although several phenotypic features overlap with those of trisomy 13 and trisomy 18, the individual structural abnormalities observed in Trisomy 9 are not sufficiently specific to establish a prenatal diagnosis based on imaging alone [2–5]. Therefore, recognition of severe multisystem involvement should prompt consideration of an underlying chromosomal abnormality and appropriate genetic testing to establish a definitive diagnosis.

A definitive prenatal genetic diagnosis can provide a basis for prognostic counseling, shared decision‐making, and advance care planning when a life‐limiting fetal condition is identified [6, 7]. Perinatal palliative care provides an interdisciplinary framework for aligning obstetric and neonatal management with the values and preferences of the family, including decisions regarding fetal monitoring, mode of delivery, neonatal resuscitation and respiratory support, family presence at birth, and comfort‐focused care [6, 7]. At our institution, family members are not routinely present in the operating room during cesarean delivery, and the neonatal care area is generally positioned separately from the mother. Such individualized planning requires coordination among obstetricians, neonatologists, clinical genetic specialists, nurses, and other healthcare professionals and may extend beyond birth and neonatal death to bereavement and psychological support for the family [6–10].

We report a prenatally diagnosed case of apparently nonmosaic Trisomy 9 to illustrate how prenatal phenotypic assessment and definitive cytogenetic diagnosis informed prognostic counseling and individualized perinatal management.

## 2. Case Presentation

A 44‐year‐old woman, gravida 4 para 1, conceived spontaneously. Her obstetric history included a previous cesarean delivery following uterine myomectomy and two spontaneous miscarriages at 8 weeks’ gestation. The current pregnancy was conceived spontaneously, and she received routine antenatal care at a local clinic. Information regarding whether aneuploidy screening or diagnostic testing had been offered before referral was unavailable. At 24 weeks’ gestation, fetal growth restriction (FGR), microcephaly, and a large ventricular septal defect (VSD) were identified, and she was referred to our hospital at 25 weeks and 2 days’ gestation for further evaluation and perinatal management. In Japan, induced abortion under the Maternal Protection Act is limited to pregnancies before 22 weeks of gestation; therefore, termination of pregnancy was not a legally available option when the fetal abnormalities were identified in this case.

Detailed fetal evaluation at our institution using ultrasonography (US) and magnetic resonance imaging (MRI) demonstrated severe multisystem abnormalities, including central nervous system abnormalities (agenesis of the corpus callosum and microcephaly); craniofacial abnormalities (low‐set ears, frontal bossing, nasal deformity, microphthalmia, and bilateral cleft lip and palate); a cardiovascular abnormality (large VSD); a renal abnormality (horseshoe kidney); and musculoskeletal abnormalities (prominent shoulders, thoracic hypoplasia, overlapping fingers, rocker‐bottom foot, and clubfoot). Amniotic fluid volume remained within the normal range throughout pregnancy. The presence of severe multisystem abnormalities suggested an underlying chromosomal disorder; however, the individual imaging findings were not considered sufficiently specific to establish a diagnosis. After counseling by a clinical genetic specialist regarding the possible chromosomal etiology and available genetic testing, the patient elected to undergo amniocentesis at 28 weeks and 3 days’ gestation. G‐banding analysis demonstrated 47,XY,+9 in all 100 examined amniotic‐fluid cell colonies, findings consistent with apparently nonmosaic Trisomy 9 (Figure 1). No postnatal cytogenetic testing using another tissue was performed.

**FIGURE 1:**
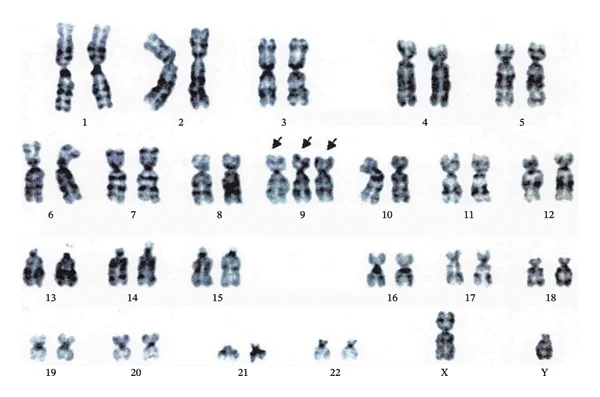
G‐banding karyotype showing 47,XY,+9 in the examined amniotic‐fluid cells, consistent with apparently nonmosaic Trisomy 9.

Following the cytogenetic diagnosis, the patient and her family received multidisciplinary counseling from obstetricians, pediatricians, and clinical genetic specialists regarding the fetal abnormalities, the extremely poor prognosis, and the expected perinatal course. Genetic counseling also addressed the cytogenetic diagnosis, its prognostic implications, recurrence considerations, and options for future pregnancies. Through shared decision‐making, the family expressed that they did not wish to pursue aggressive life‐prolonging treatment but strongly wished to have an opportunity to meet and spend time with their infant alive. Accordingly, an individualized birth plan was established before delivery. Cesarean delivery had already been planned because of her previous cesarean delivery and history of uterine myomectomy. In accordance with the family’s wishes, fetal heart rate monitoring and cesarean delivery for fetal indications were included in the birth plan to maximize the possibility of live birth. For neonatal resuscitation, mask ventilation was planned if required, whereas no further resuscitative or life‐prolonging interventions were planned. The plan also prioritized family presence at delivery and immediate postnatal contact, allowing the infant to remain with the family after birth.

At 36 weeks and 2 days’ gestation, nonreassuring fetal status developed during fetal heart rate monitoring, and emergency cesarean delivery was performed in accordance with the previously established birth plan. The husband was permitted to be present in the operating room. To maintain maternal safety and surgical sterility, a sterile drape was used to shield the operative field from direct view, and the neonatal care area was positioned close to the patient and her husband to facilitate immediate family contact after birth (Figure 2).

**FIGURE 2:**
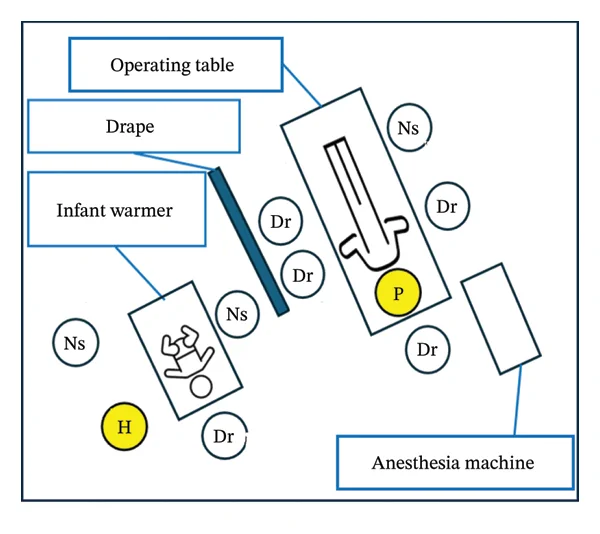
Operating room arrangement for individualized family‐centered care during cesarean delivery. In contrast to our usual cesarean delivery practice, the husband was allowed to be present, and the neonatal care area was positioned close to the patient to facilitate immediate family contact after birth while maintaining surgical sterility. Dr, doctor; Ns, nurse; P, patient; H, husband. The schematic illustration was created by the authors using Microsoft PowerPoint (Microsoft Corporation, Redmond, WA, USA).

The male neonate was born small for gestational age, weighing 1198 g (−4.3 SD), with Apgar scores of 0 and 5 at 1 and 5 min, respectively. He exhibited characteristic dysmorphic features, including microphthalmia, a bulbous nose, bilateral cleft lip and palate, frontal bossing, microtia, low‐set ears, and prominent shoulders. At birth, he had no detectable heart rate or spontaneous respiration. In accordance with the birth plan, mask ventilation with 100% oxygen was initiated. Approximately 2 min after birth, his heart rate became detectable, and weak crying was observed. Mask ventilation was discontinued after spontaneous respiration was established, and no further resuscitative intervention was performed. He was then brought to his parents for immediate contact. After the family was informed of the options of respiratory support in the neonatal intensive care unit or remaining together without further life‐prolonging treatment, they chose the latter. The infant remained with his family and died approximately 9 h after birth in their presence. The patient initially expressed strong appreciation for the care she had received and reported satisfaction with the birth experience.

At 1 month postpartum, the patient developed persistent depressive symptoms and was referred for psychiatric evaluation, where she was diagnosed with adjustment disorder. She subsequently required long‐term psychiatric follow‐up and was diagnosed with depression approximately 1 year after delivery.

## 3. Discussion

This case highlights the clinical importance of prenatal recognition of severe multisystem fetal abnormalities and definitive cytogenetic diagnosis of apparently nonmosaic Trisomy 9. The genetic diagnosis enabled phenotype‐informed prognostic counseling and provided the basis for individualized perinatal management.

Prenatal diagnosis of nonmosaic Trisomy 9 is clinically important, as it provides information for prognostic counseling and facilitates advance care planning [2, 6]. Both nonmosaic and mosaic forms of Trisomy 9 are associated with heterogeneous multisystem abnormalities, including FGR; craniofacial abnormalities, such as microphthalmia and low‐set ears; central nervous system abnormalities, including ventriculomegaly and agenesis of the corpus callosum; congenital heart defects, such as VSDs; genitourinary abnormalities, including renal malformations; and skeletal abnormalities, such as overlapping fingers and clubfoot [1, 2, 11]. Recent reports have further delineated the phenotypic spectrum of nonmosaic Trisomy 9. Xu et al. reviewed 59 reported cases of nonmosaic Trisomy 9 [2], and Verscaj et al. more recently further characterized the clinical phenotype of nonmosaic Trisomy 9 [12]. The findings in the present case, including microphthalmia, low‐set ears, cleft lip and palate, VSD, horseshoe kidney, overlapping fingers, and clubfoot, are broadly consistent with the currently recognized phenotypic spectrum [2, 3, 12]. Although the phenotypic spectrum substantially overlaps between the two forms, nonmosaic Trisomy 9 is typically associated with a severe phenotype and perinatal mortality, whereas mosaic Trisomy 9 shows marked phenotypic variability, ranging from severe prenatal abnormalities to milder phenotypes in long‐term survivors [1, 2].

The severe prenatal multisystem phenotype in the present case, including marked FGR and central nervous system, craniofacial, cardiac, renal, and musculoskeletal abnormalities, was consistent with the severe phenotypic spectrum reported in nonmosaic Trisomy 9 and with the poor neonatal outcome observed in this case. However, these abnormalities are not specific to Trisomy 9 and overlap with those observed in other chromosomal disorders [2–5]. Therefore, the value of detailed phenotypic assessment is not that any individual finding can establish a prenatal diagnosis of Trisomy 9 but rather that recognition of a pattern of severe multisystem involvement can prompt appropriate genetic testing and subsequently contribute to phenotype‐informed prognostic counseling once the cytogenetic diagnosis has been established.

Cytogenetic characterization is particularly important because the phenotypic severity and survival outcomes of affected individuals vary according to the cytogenetic form of Trisomy 9 [1, 2]. Nonmosaic Trisomy 9 is generally associated with an extremely poor prognosis, with affected pregnancies frequently resulting in spontaneous abortion, intrauterine fetal death, or death during the early neonatal period. In contrast, individuals with mosaic Trisomy 9 may survive beyond infancy and, in some cases, into childhood or adulthood [1, 2]. These differences underscore the importance of cytogenetic characterization for accurate prognostic counseling and individualized perinatal care planning. In the present case, Trisomy 9 was identified in all 100 examined colonies, with no normal cell line detected, strongly supporting a diagnosis of apparently nonmosaic Trisomy 9 in the examined amniotic‐fluid specimen. However, because cytogenetic analysis was limited to amniotic‐fluid cells and no postnatal analysis of another tissue was performed, very low‐level or tissue‐limited mosaicism elsewhere in the fetus cannot be completely excluded. Given the extremely poor prognosis associated with the nonmosaic form of Trisomy 9, establishing a definitive prenatal diagnosis provided clinically relevant information for prognostic counseling, shared decision‐making, and subsequent individualized perinatal care planning. Genetic counseling also addressed the presumed mechanism of nonmosaic Trisomy 9, recurrence risk, and implications for future pregnancies.

In our case, based on the definitive prenatal cytogenetic diagnosis of Trisomy 9, with findings consistent with an apparently nonmosaic form, multidisciplinary counseling and shared decision‐making enabled the family and healthcare professionals to develop an individualized perinatal care plan. In pregnancies complicated by life‐limiting fetal conditions, perinatal management is generally directed toward minimizing maternal risk while respecting parental values. Perinatal palliative care does not necessarily imply the uniform avoidance of fetal monitoring or operative delivery; rather, these decisions should be individualized according to maternal considerations and informed parental goals [6, 7]. Current guidelines also emphasize individualized, patient‐centered decision‐making that incorporates parental values and preferences [6]. In the present case, the family wished to avoid aggressive life‐prolonging treatment while strongly prioritizing the opportunity to meet and spend time with their infant alive. Cesarean delivery had already been planned because of the previous cesarean delivery and history of uterine myomectomy. In addition, fetal heart rate monitoring and cesarean delivery for fetal indications were explicitly incorporated into the birth plan to maximize the possibility of live birth. Consequently, when nonreassuring fetal status developed at 36 weeks and 2 days, emergency cesarean delivery was consistent with, rather than contrary to, the previously established palliative care plan. The management described in this case reflects the core principles of perinatal palliative care, including multidisciplinary counseling, shared decision‐making, respect for parental values, and facilitation of family bonding.

The neonatal care plan was similarly individualized in advance. Mask ventilation was planned if required, whereas no further resuscitative or life‐prolonging interventions were planned. At birth, mask ventilation was initiated because the infant had no detectable heart rate or spontaneous respiration. After spontaneous respiration was established, ventilation was discontinued, and no further resuscitative intervention was performed. The infant was then brought to his parents and remained with his family until death. Thus, the brief respiratory intervention was not intended to pursue prolonged survival but was consistent with the previously agreed goal of facilitating the family’s opportunity to meet and spend time with their infant alive [6, 7].

In the present case, the operating room arrangement was modified to allow the husband to be present and to position the neonatal care area close to the mother. These modifications facilitated immediate postnatal contact between the infant and the family while maintaining maternal safety, surgical sterility, and appropriate neonatal care (Figure 2). This illustrates how advance care planning can be translated into practical, system‐level accommodations as part of individualized family‐centered perinatal palliative care [6, 7].

The subsequent development of adjustment disorder 1 month postpartum and depression approximately 1 year after delivery, despite initial satisfaction with the birth experience, illustrates that satisfaction with perinatal care and subsequent psychiatric morbidity are not mutually exclusive. Her history of two previous early pregnancy losses represents relevant psychosocial background; however, its relationship to her subsequent psychiatric course cannot be determined from this case. Similarly, a single case cannot establish either a protective or causal relationship between the individualized birth plan and subsequent psychological outcomes. Perinatal loss is associated with substantial risks of adverse psychological outcomes, which may persist beyond the immediate bereavement period [8, 9, 13]. Therefore, bereavement and psychological support should remain available longitudinally after neonatal loss [8–10, 13].

In conclusion, this case highlights the importance of definitive prenatal cytogenetic diagnosis of Trisomy 9, with findings consistent with an apparently nonmosaic form, as a basis for an individualized multidisciplinary care pathway extending from prognostic and genetic counseling to shared decision‐making, advance birth planning, neonatal palliative care, family‐centered care at birth and death, and longitudinal psychological support after neonatal loss. Detailed phenotypic assessment remains important for recognizing severe multisystem involvement and prompting appropriate genetic testing, although the individual findings are not specific to Trisomy 9. This case also emphasizes that individualized perinatal care should remain responsive to family goals while recognizing that supportive needs may continue long after neonatal death.

## Author Contributions

Hitoshi Isohata: conceptualization and writing–original draft.

Yoshihiro Yoshimura, Yu Yamazaki, Hiroyuki Goto, Kyoko Hattori, and Takao Shimaoka: writing–review and editing.

Fumio Takada: supervision and writing–review and editing.

Daigo Ochiai: conceptualization, supervision, and writing–review and editing.

## Funding

The authors received no specific funding for this work.

## Ethics Statement

Ethical approval was not required for a single case report.

## Consent

Written informed consent was obtained for publication of the clinical details, prenatal and postnatal images, neonatal outcome, and maternal psychological follow‐up presented in this report. Potentially identifiable information was handled appropriately to protect the privacy of the patient and her family.

## Conflicts of Interest

The authors declare no conflicts of interest.

## Data Availability

The data supporting the findings of this case report are available from the corresponding author upon reasonable request. Additional data are not publicly available because they contain information that could compromise patient privacy.
